# Herbal and Alternative Medicine Use in Tanzanian Adults Admitted with Hypertension-Related Diseases: A Mixed-Methods Study

**DOI:** 10.1155/2017/5692572

**Published:** 2017-05-28

**Authors:** Anthony Liwa, Rebecca Roediger, Hyasinta Jaka, Amina Bougaila, Luke Smart, Stacey Langwick, Robert Peck

**Affiliations:** ^1^Department of Clinical Pharmacology, Weill Bugando School of Medicine, Catholic University of Health and Allied Sciences, P.O. Box 1464, Mwanza, Tanzania; ^2^Department of Internal Medicine, Mount Sinai Hospital, 17 East 102nd St, 7th Floor, New York, NY 10029, USA; ^3^Department of Internal Medicine, Weill Bugando School of Medicine, Catholic University of Health and Allied Sciences, P.O. Box 1464, Mwanza, Tanzania; ^4^Weill Cornell Medical College in Qatar, Doha, Qatar; ^5^Center for Global Health, Department of Medicine, Weill Cornell Medical College, New York, NY, USA; ^6^Department of Anthropology, Cornell University, 261 McGraw Hall, Ithaca, NY, USA

## Abstract

**Background:**

Hypertension is increasingly common in sub-Saharan Africa where traditional medicine use is also common. We conducted a hospital-based, mixed-methods study to determine prevalence, pattern, and correlates of herbal and alternative medicine use in Tanzanian adults hospitalized with hypertension.

**Methods:**

A standardized questionnaire was administered. In-depth interviews were performed on a subset of participants. Factors associated with herbal medicine use were determined by logistic regression. The association between traditional medicine uses and allopathic medication adherence was determined using ordinal logistic regression. Qualitative data were analyzed according to grounded theory.

**Results:**

Of 213 adults enrolled, 52 (24.4%) reported using herbs during the previous month and 47 (22.1%) reported concurrent use of herbs and allopathic medicines. Lower educational level, nonprofessional employment, and lack of health insurance were significantly associated with herbal medicine use. Alternative medicines use was not associated with lower medication adherence. Qualitative interviews identified several important themes including reasons for herbal medicine use.

**Conclusion:**

The use of traditional medicines is very common among patients with hypertension. Adults from low socioeconomic status, those with misunderstandings about hypertension, and those without health insurance were more likely to take herbs. Open, nonjudgmental communication between healthcare workers and patients regarding use of traditional medicines must be encouraged in Africa.

## 1. Background

The burden of hypertension is increasing in sub-Saharan Africa (SSA) [1], with an age-standardized prevalence of ~40% in adults over 25 years of age [2]. Of adults with hypertension in Tanzania, only ~35% are aware of their diagnosis, ~5% are on treatment, and ~1% are controlled [3]. At our own hospital in Tanzania, hypertension is the second most common diagnosis on the medical wards accounting for >15% of all admissions, hospital days, and deaths [4].

WHO has defined “traditional medicine” as sum total of the knowledge, skills, and practices based on the theories, beliefs, and experiences indigenous to different cultures, whether explicable or not, used in the maintenance of health, as well as in the prevention, diagnosis, improvement, or treatment of illnesses. “Herbal medicines,” on the other hand, in a more specific term include herbs, herbal materials, herbal preparations, and finished herbal products that contain active ingredients parts of plants. In the current study we used terms “herbal” to describe traditional medicine that is derived from plants and “alternative medicine” to describe other traditional medicines.

Traditional medicine use in hypertension and its related diseases can lead to adverse outcome, related to delay in seeking proper treatment, leading to increased severity or complications of the disease, drug interactions, and poor adherence to allopathic medicines that are known to control the disease [5]. Although traditional medicine use is common in sub-Saharan Africa (SSA) [5–9], little is known about its overlap with hypertension. In our own, recent meta-analysis, we found only 4 published studies reporting data regarding herbal and alternative medicine use in adults with hypertension in Africa. These 4 studies indicated that 25–65% of adults with hypertension reported use of herbal and alternative medicines [10].

To the best of our knowledge, no published data exists regarding the pattern of herbal and alternative medicine use among adult inpatients or the association between herbal and alternative medicine use and antihypertensive medications adherence. Specifically, it determined (1) the prevalence and pattern of herbal and alternative medicine utilization, (2) factors associated with herbal medicine use, and (3) whether herbal or alternative medicine use was associated with medication nonadherence. We hypothesized that herbal and other alternative medicine use is common among Tanzanians hospitalized with hypertension-related diseases and is associated with nonadherence to allopathic medicines.

## 2. Methods

### 2.1. Study Setting

This study was conducted in the adult medical wards of Bugando Medical Centre (BMC). BMC is a tertiary and teaching hospital that serves the Lake region in the northwestern part of mainland Tanzania.

### 2.2. Study Design and Recruitment of Participants

To provide better understanding of the problem of herbal and other alternative medicines use, the current study was conducted prospectively by a hospital-based mixed quantitative and qualitative research design and was conducted concurrently between April and October 2014. The subjects for this study were selected from patients hospitalized at the medical wards of BMC. All adults (>18 years of age) admitted with hypertension-related diagnoses were approached within 24 hours of admission and invited to participate in our study.

Blood pressure measurement was done at least twice (once on each arm) after 5 minutes of sitting using a mercury sphygmomanometer. If either or both first 2 blood pressure measurements are elevated, a 3rd blood pressure measurement was taken and the average of the 2nd and 3rd measurements was considered for the diagnosis of hypertension.

Diagnoses of hypertensive diseases, that is, hypertensive stroke, hypertensive heart failure, or hypertensive renal failure, were made per WHO International Statistical Classification of Diseases and Related Health Problems 10th Revision (ICD-10) version for 2010. Adults with altered mental status were excluded since they could not provide adequate or reliable information.

### 2.3. Data Collection and Analysis

A structured researcher-administered questionnaire with both closed ended and open-ended questions was used to collect quantitative data from eligible patients. The questionnaire consisted of sections that assessed patient information such as age, gender, marital status, education level, and duration of disease. Responses on patients' knowledge about hypertension, use of herbal and other alternative medicines, reasons for herbs use, sources of herbs, and adherence to antihypertensive medications were obtained.

Adherence to antihypertensive medications was measured using a Morisky Medication Adherence Scale, the 4-item scale (MMAS-4). MMAS-4 is a self-reported commonly used adherence-screening tool. It is composed of 4 yes/no questions about past medication use patterns and is validated in antihypertensive patients in several countries [11, 12].

Data were coded and entered into Microsoft Excel 2010 software (Microsoft Corporation, Redmond, WA, USA) and analyzed using STATA version 12 (StataCorp, Collage Station, Texas). Categorical variables were described as proportions (percentiles) and continuous variables were described as medians [interquartile ranges]. Differences between patient groups were calculated along with 95% confidence intervals (CI). To examine potential factors associated with the current use of herbal and alternative medicines, univariate and multivariate logistic regression were conducted. Factors associated with adherence to allopathic antihypertensive medications were explored using ordinal logistic regression. The Brant test was used to assess the proportional odds assumption. A two-sided* p* value < 0.05 was considered significant. Since this was an exploratory analysis, we did not make any adjustment for multiple comparisons.

12 study subjects who used herbal or other alternative medicines were selectively chosen and underwent in-person, semi-structured interviews. The primary investigator conducted these interviews in a private space on the medical wards and they were ~20 minutes long. Interviews were conducted in Kiswahili, recorded, transcribed, and then translated into English by a professional translation service.

The analysis process had two levels. We started collecting qualitative data through open-ended questions. As we reviewed the data collected, repeated ideas, concepts, or elements become apparent and were eventually tagged with inductive and deductive* codes*, which were extracted from the data. The second level involved collection of data, and as data were rereviewed, codes were grouped into themes and then into categories. These categories were the basis for new theory. Two independent researchers (AL and RP) identified themes and supporting quotations were selected.

In-depth interviews were performed on 12 adults admitted with hypertension. After breaking data into conceptual components, these concepts were theorized, integrated, and refined, and four themes/theories emerged: (i) lack of understanding about hypertension, (ii) the exact identity of herbal remedies being often unknown, (iii) contradictory opinions on risks of concurrent use of allopathic and herbal medications, and (iv) 3 common reasons for herbal medicine use.

### 2.4. Ethical Approval

Ethical approval for the study was obtained from the Bugando Research Ethics Committee in Tanzania and the Institutional Review Board of Weill Cornell Medical College. Each participating patient provided written informed consent. Consent to participate in this study did not affect the clinical care of the patients.

## 3. Results

### 3.1. Enrollment

Between April 1 and October 1, 377 adults were hospitalized to the medical wards of Bugando Medical Centre with hypertension-related diseases. Of these, 164 were excluded for the following reasons: 147 were unable to give a reliable history due to altered mental status (mostly due to stroke or hypertensive encephalopathy), 9 declined to participate in the study, and 8 were discharged within 24 hours (before enrollment could occur). The remaining 213 adults consented and were enrolled.

### 3.2. Demographic Characteristics

Among the 213 adult inpatients enrolled in our study, 112/213 (52.6%) were female, the median age was 56 years [45–67], and only 34/213 (16.0%) had completed secondary school education. Most study subjects (162/213 (76.1%)) were aware of their hypertension before admission but 51/213 (23.9%) were newly diagnosed to have hypertension during the current hospital admission. Most (159/213 (74.7%)) had a previous history of using antihypertensive medications but only 103/213 (48.4%) was taking medications before hospitalization. Of the 159 study subjects who reported prior use of antihypertensive medications, 35/159 (22.0%) reported high adherence, 38/159 (23.9%) reported medium adherence, and 86/159 (54.1%) reported low adherence to antihypertensive medications. The average years of patient illness were 5 years [2–5, 10–14]. Most (173/213 (81.2%)) had at least 1 comorbid condition: 54/173 (24.5%) had heart failure, 39/173 (18.4%) had diabetes mellitus, 24/173 (11.3%) had kidney disease, and 20/173 (9.4%) reported a history of stroke. The characteristics of the study population are presented in Table 1.

### 3.3. Outcomes

Of the 213 Tanzanian adults in patients with hypertension that participated in the study, 161/213 (75.6%) reported using herbal and alternative medicines at some point in their life. 69/213 (32.4%) used herbal and alternative medicines specifically to manage hypertension at some point in their life and 52/213 (24.4%) used herbal and alternative medicines in the past month before admission to the hospital. 38/213 (17.8%) of the respondents admitted to having stopped allopathic medications for herbal and alternative medicines and 47/213 (22.1%) reported to the concurrent use of herbal and alternative medicines and allopathic medications. Only 20/213 (9.4%) reported having been asked about traditional medicine use by their admitting doctor. Herbal and other alternative medicines use did not differ significantly by gender. The other outcomes related to the utilization of herbal and alternative medicine are presented in detail in Table 2.

### 3.4. Factors Associated with Herbal Medicine Use

Table 3 displays the results of our logistic regression analysis to determine factors associated with herbal medicine use for hypertension in the past month before admission. Patients with higher categories of education (secondary education to college education) were less likely to consume herbal medicine compared to their counterparts (*p* < 0.007; OR = 0.49; CI 0.29–0.82). Professionals (salaried workers), businessmen (business owners), and government employees were also less likely to consume herbal medicine compared to farmers (*p* = 0.002; OR = 0.27; CI 0.11–0.61). Study subjects with medical insurance were also less likely to use herbal medicines (*p* = 0.001; OR = 0.24; CI 0.10–0.57). Stroke was the only comorbid condition that was significantly associated with herbal medicine use (*p* = 0.008; OR = 3.60; CI 1.40–9.21).

### 3.5. Factors Associated with Allopathic Medication Adherence

Table 4 displays our ordinal logistic regression analysis of factors associated with poor medication adherence. Higher education level (secondary school and above) was associated with better medication adherence (*p* = 0.04; OR = 0.63; CI 0.40–0.99 for lower adherence). Study subjects who believed that hypertension could not be cured with a short course of traditional or allopathic medicines also had better medication adherence (*p* = 0.001; OR = 0.34; CI 0.18–0.63 for low adherence). In addition, study subjects who were diagnosed with hypertension in places other than a health facility were less likely to be adherent to the antihypertensive medications (*p* = 0.02; OR = 3.80; CI 1.21–11.85). Of note, neither herbal and alternative medicine use nor attendance to traditional healers was significantly associated with nonadherence. In fact, none of the variables for herbal and alternative medicine utilization were significantly associated with nonadherence.

### 3.6. Herbal Remedies and Other Alternative Therapies

Among the 161 patients who reported to use herbal and alternative medicine, 134/161 (83.2%) did not know the names of the herbs that they were consuming. Of the 27/161 (16.8%) patients who knew the names of the herbs that they were taking, 21/161 (13.0%) patients mentioned taking more than one herbal preparation. The most frequent remedies that were mentioned include* garlic* (11),* ginger *(3)* honey *(2),* carrots (2), avocado seeds* (2),* bit roots *(1),* wood charcoal *(1),* papaya seeds *(1),* lemons *(1),* onions *(1), and* aloe vera* (1). Other patients mentioned plant derived remedies known by tribal names including Mronge seeds* (Moringa oleifera)* (2), Kuwarumbizo (Ikizu tribe) (1), Engeni tree (1), and Nyabururu (Sukuma tribal name) (1). Four (4) study subjects reported using “Chinese medicines” but were not able to give specific names. Some patients also mentioned commercially available remedies/products including “Herbal Works” (1) and “Forever Living” health products (1).

### 3.7. Reasons for Herbal Medicine Use

The 69 study subjects who had used herbal medicines specifically for the treatment of hypertension were asked to provide reasons for herbal medicine use: 22/69 (31.9%) reported using herbal medicines because they still believed in traditional ways of treatment and customs of healthcare; 12/69 (17.4%) were convinced by friends or people who have claimed to have been cured by herbs; 11/69 (15.9%) were looking for other options for treatment; 7/69 (10.2%) reported pressure from a spouse or family member; 5/69 (7.3%) reported that they did not want to go to hospital because of uncaring attitude of hospital staff; 3/69 (4.4%) patients used herbal medicines because they did not have funds to go to hospital; 3/69 (4.4%) patients wanted to lose weight; 2/69 (2.9%) patients were convinced by radio advertisement; 2/69 (2.9%) reported to have been bewitched to think traditional ways were the better option; 1/69 (1.5%) reported that he was previously cured of hypertension with herbs; and 1/69 (1.5%) was advised by a local healer who knew the herbs.

### 3.8. Qualitative Analysis

In-depth interviews and the following themes emerged: (i) lack of understanding about hypertension, (ii) the exact identity of herbal remedies being often unknown, (iii) contradictory opinions on risks of concurrent use of allopathic and herbal medications, and (iv) 3 common reasons for herbal medicine use.

#### 3.8.1. Lack of Understanding about Hypertension

Participants in this study consistently expressed a limited understanding of hypertension and reported having received very little education about this condition:

I do not know what it is, except that they have tested me and they said I have high blood pressure. (Male, 42 years)

#### 3.8.2. Exact Identity of Herbal Remedies Being Often Unknown

There are many forms in which herbal medicines were prepared, the most common of which were extracts from plants which were either diluted or boiled to obtain its liquid form through steam. Whole herb consumption was also practiced either fresh or in dried form.

What I know it is a tree, but I do not know what kind of a tree… I found that the stuff has already been pounded… you just mix it with water and drink it… they put one, two or even five kinds of those trees. (Female, 48 years)

#### 3.8.3. Contradictory Opinions on Risks of Concurrent Use of Allopathic and Herbal Medications

Participants expressed differing opinions regarding whether it is acceptable to take both allopathic and herbal medications at the same time:

There is a problem… you cannot mix medications because if you mix you will not know what is making you feel better. (Male, 55 years)

Yes you take them [herbs] as well except that you must know your time. (Male, 42 years)

#### 3.8.4. 3 Common Reasons for Herbal Medicines Use

Interest in herbal medicines was commonly attributed to 3 main reasons: (i) lack of funds to go to hospital, (ii) pressure from relatives and friends, and (iii) the desire for a complete cure:

Sometimes you do not have money…. now someone comes and say that I was helped by avocado seeds and you see it's free you prepare it to obtain good health. (Female, 50 years)

If you are sick, the advice come from any human being… because you are sick, you do not have a choice… if someone come and tell you that I have been helped by this certain tree, you go and dig it up. (Female, 49 years)

They said that they treat diseases that cannot be cured in hospitals by using traditional medicines. (Male, 65 years)

## 4. Discussion

Herbal and other alternative medicines use is highly prevalent among Tanzanian adults admitted with hypertension-related conditions and many of those adults were using herbs specifically for hypertension. More than 75% of the adults in our study reported using herbal medicines in the past and nearly 70% had attended a traditional healer. Approximately 25% of adults were currently using herb specifically for management of hypertension. This study is the first to describe the pattern of herbal and alternative medicine in African inpatients. This pattern is broadly similar to findings from hypertension clinics in South and West Africa [6, 7, 13, 14], as well as community-based studies in Eastern and Western African countries [8, 9]. In all of these settings, approximately one-quarter of African adults with hypertension reported the use of traditional medicines and most of these were using herbal medicines. Because herbal medicines in particular are such an important part of hypertension self-management throughout Africa, collaboration with traditional healers should be explored as a possible route for improving hypertension care [9].

In the current study, nearly 20% of adults reported stopping their allopathic medicines for herbal medicine and >20% were concurrently using both herbal and allopathic medications. More than 70% of patients believed that there is no problem to use herb together with allopathic medications. These findings are concerning. Although some of the herbs used by our study subjects are likely harmless, most patients did not know what they were taking, and concurrent use of some herbs could hamper the effectiveness of allopathic antihypertensive drugs such as the interaction between St. John's Wart and calcium channel blockers or the interaction between garlic and thiazide diuretics [15, 16]. Interactions between herbs and cardiovascular drugs could also increase the toxicological effects of cardiovascular drugs such as the interaction between garlic and warfarin or the interaction between* Ginseng* and Digoxin [5, 17, 18]. Sodium-sparing herbal aquaretics such as dandelions, for example, may offset antihypertensive effects of thiazide diuretics [16]. The use of* ginseng* with digoxin is associated with increased digoxin concentration in the blood and hence digoxin toxicity [19]. African adults with hypertension who are using herbs clearly require counseling not to stop their allopathic medicines for herbs as well as counseling as to possible interactions between the herbs they are using and their cardiovascular medications.

Factors that were significantly associated with the use of herbal medicines for hypertension included lower education level, nonprofessional occupation, lack of medical insurance, and history of comorbid stroke. The lower education level was also found to be associated with the use of herbal medicines in Nigeria [14]. Occupation has also been shown to be associated with herbal medicine use in another study from West Africa [9]. It is possible, though, that individuals of higher education level and occupation status are simply less willing to report herbal medicine use and this deserves further investigation. Inability to afford allopathic medicines was associated with herbal medicine use in Ghana [6]. The association between herbal medicine use and stroke may be related to the cultural perception in Tanzania that stroke may have an extra-biological (i.e., “magical”) origin, a perception that has been associated with herbal medicine use in other studies [9]. These data indicate populations for whom more intensive counseling should be provided regarding herbal medicines. Providing insurance may have a beneficial effect.

Among patients who were aware of the herbal preparations that they are taking, garlic was the most frequently used herb. Garlic* (Allium sativum)* has been closely studied. Garlic has demonstrated multiple cardiovascular benefits that include lowering blood pressure, inhibiting platelet aggregation, enhancing fibrinolytic activity, and reducing cholesterol and triglyceride levels. However, despite all this evidence the routine use has not been advised because of methodological shortcomings of the published literature [20] and extreme caution is advised if garlic is taken concomitantly with CYP2E1 substrates [15] or in patients taking warfarin [16]. Studies are needed to determine possible benefits and harms of native African herbs that are used by traditional healers for hypertension.

Over 90% of study subjects reported that their doctors did not ask them about the use of either herbs or other alternative therapies for hypertension. These findings are consistent with other studies from West Africa which indicate (1) that the overwhelming majority of healthcare workers are not aware of the herbal medicine use of the hypertensive adults in their care [6, 14] and (2) that a leading reason for this is fear and lack of inquiry of the part of the healthcare worker [6].

In our study, current use of traditional medicines for hypertension was not associated with lower compliance to allopathic medicines, as it has been in prior studies [6, 13]. It is therefore important that doctors not only ask about herbs and other alternative therapies among adults admitted with hypertension but also provide careful, specific, and nonjudgmental recommendations. The authors suspect that more patients will be willing to listen and follow advice if clinicians apply this careful, discerning approach. Further studies are needed to determine the best way to ask and educate about herbal medicine use in the African context.

## 5. Conclusion

The use of herbs and other alternative therapies are very common among patients with hypertension. Nearly a quarter of the hypertensive adults in our study reported recently taking herbs specifically for the treatment of hypertension. Adults from low socioeconomic status, those with misunderstandings about hypertension, and those without insurance were more likely to take herbs. Study subjects often chose to take herbs due to poverty, family pressure, and a desire for a complete cure. Use of herbal or alternative therapies was not associated with noncompliance. Their doctors had asked very few patients about herbal medicine use. Open, nonjudgmental communication between healthcare workers and patients regarding use of herbs and other alternative medicines must be encouraged in Africa. Future investigations should focus on identifying the magnitude of the problem in the community level, traditional healer's knowledge about hypertension, and the timing of initiation of herbal use and alternative therapies.

## Figures and Tables

**Table 1:** Sociodemographic and clinical characteristics of 213 consecutive adults hospitalized at Bugando Medical Centre with hypertension-related diseases.

Variable	Value (*n* = 213)
Proportion (%)	
Median [IQR]	
Female gender	112 (52.6)
Age (years)	56 [45–67]
Education level	
** **Not completed primary school	58 (27.2)
** **Completed primary school	121 (56.8)
** **Completed secondary school	34 (16.0)
Occupation	
** **Farmer	78 (36.6)
** **Small scale business	54 (25.4)
** **Professional, business, or student	81 (38.0)
Health insurance	70 (32.9)
Hypertension diagnosis	
** **New diagnosis	51 (23.9)
** **Previously diagnosed	162 (76.1)
Medication adherence (MMAS-4 score)^**∗**^	
** **High (0)	35 (22.0)
** **Medium (1-2)	38 (23.9)
** **Low (3-4)	86 (54.1)
Systolic blood pressure	160 [150–180]
Diastolic blood pressure	100 [90–120]
Is hypertension curable (or is it a lifelong condition)?	
** **No (hypertension is a lifelong condition)	95 (44.6)
** **Yes (hypertension is curable)	118 (55.4)
Is there anything that can be done to prevent hypertension?	
Yes	54 (25.4)
No	159 (74.7)
Years since hypertension diagnosis	5 [2–10]
Place of diagnosis	
Hospital	194 (91.1)
Other	19 (8.9)
History of hypertensive medication	159 (74.7)
Currently on hypertensive medication	103 (48.4)
Prevalence of comorbid conditions	173 (81.2)
Heart failure	54 (24.5)
Diabetes mellitus	39 (18.4)
Kidney disease	24 (11.3)
Stroke	20 (9.4)

^*∗*^MMAS-4, Morisky Medication Adherence Scale, the 4-item scale; IQR, interquartile range.

**Table 2:** Herbal medicine utilization patterns in 213 consecutive adults hospitalized to Bugando Medical Centre with hypertension-related diseases.

Outcome	Total (*n* = 213)	Female (*n* = 112)	Male (*n* = 101)	*p* value
Proportion (%)				
Median [IQR]				
Used herbal medicine in last month^*∗*^	52 (24.4)	26 (23.2)	26 (25.7)	0.67
Ever attended a traditional healer for any reason	144 (67.6)	77 (68.8)	67 (66.3)	0.71
Ever attended traditional healer for hypertension	59 (27.7)	29 (25.9)	30 (29.7)	0.54
Ever used herbal medicine for any reason	161 (75.6)	35 (31.3)	74 (73.3)	0.46
Ever used herbs for hypertension	69 (32.4)	38 (33.9)	31 (30.7)	0.61
Practiced religious healing for hypertension	30 (14.1)	17 (15.2)	13 (12.9)	0.63
Using herbal medicine in hospital	10 (4.7)	5 (5.0)	5 (4.5)	0.87
Stopped allopathic medicines for herbal medicine	38 (17.8)	17 (15.2)	21 (20.8)	0.29
Used herbal medicine with allopathic medicines	47 (22.1)	28 (25.0)	19 (18.8)	0.28
Believes it is okay to use herbal medicine with allopathic medicines	152 (71.4)	76 (67.9)	76 (75.3)	0.24
Traditional healer has asked about allopathic medicines	20 (9.4)	8 (7.1)	12 (11.9)	0.24
Doctors have asked about herbal medicines	20 (9.4)	11 (9.8)	9 (8.9)	0.82

^*∗*^Primary study outcome; IQR, interquartile range.

**Table 3:** Characteristics and factors associated with the current use of herbal medicine in the month prior to hospital admission.

Variable	Herbal users (*n* = 52)	Herbal nonusers (*n* = 161)	Odds ratio [95% CI]	*p* value
Proportion (%)				
Median [IQR]				
Female gender	26 (23.2)	86 (76.8)	0.87 [0.47–1.63]	0.67
Age category (years)				
≤45	13 (21.7)	47 (78.3)	1	
46–55	12 (30.0)	28 (7.0)	1.55 [0.62–3.86]	
56–65	11 (20.1)	43 (79.6)	0.93 [0.37–2.28]	
>65	16 (27.1)	43 (72.9)	1.35 [0.58–3.12]	0.65
Education level				
Not completed primary school	21 (36.2)	37 (63.8)		
Completed primary school	27 (22.3)	94 (77.7)		
Completed secondary school	4 (11.8)	30 (88.2)	0.49 [0.29–0.82]	0.007
Occupation				
Farmer	25 (32.1)	53 (68.0)	1	
Small scale business	18 (33.3)	36 (66.7)	1.06 [0.51–2.21]	
Professional, business, or student	9 (11.1)	72 (88.9)	0.27 [0.11–0.61]	0.002
Health insurance				
No insurance	45 (31.5)	98 (68.5)	1	
Insurance	7 (10.0)	63 (90.0)	0.24 [0.10–0.57]	0.001
Hypertension diagnosis				
New diagnosis	13 (25.5)	38 (74.5)	1	
Previously diagnosed	39 (24.1)	123 (75.9)	0.93 [0.45–1.92]	0.84
Years of diagnosis				
≤1	16 (32.0)	34 (38.0)		
2–4	7 (16.7)	35 (83.3)	0.43 [0.16–1.16]	
5–9	8 (28.6)	20 (71.4)	0.85 [0.31–2.34]	
≥10	21 (24.4)	72 (77.4)	0.62 [0.29–1.34]	0.34
Systolic blood pressure				
≤140	8 (18.2)	36 (81.8)		
141–160	17 (27.9)	44 (72.1)	1.74 [0.67–4.50]	
161–180	16 (29.6)	38 (70.4)	1.89 [0.72–4.97]	
>180	11 (20.4)	43 (79.6)	1.15 [0.42–3.17]	0.45
Currently on hypertensive medication	38 (23.9)	121 (76.1)	1.53 [0.81–2.89]	0.77
Is hypertension curable (or is it a lifelong condition)?				
No (hypertension is a lifelong condition)	34 (28.8)	84 (71.2)		
Yes (hypertension is curable)	18 (19.0)	77 (81.1)	0.58 [0.30–1.11]	0.098
Is there anything that can be done to prevent hypertension?				
Yes	8 (14.8)	46 (85.2)		
No	44 (27.7)	115 (72.3)	0.46 [0.19–1.04]	0.06
Place of diagnosis				
Hospital	47 (24.2)	147 (75.8)		
Other	5 (26.3)	14 (73.7)	1.12 [0.38–3.27]	0.34
MMAS Question 1 (ever forget to take medicines)	5 (12.5)	35 (87.5)	0.42 [0.14–1.26]	0.12
MMAS Question 2 (have problems remembering to take medicines)	5 (14.3)	30 (85.7)	0.54 [0.18–1.63]	0.28
MMAS Question 3 (when feel better stops taking medicines)	12 (21.8)	43 (78.2)	1.21 [0.46–3.18]	0.70
MMAS Question 4 (stop taking medicines when feeling worse with medicines)	1 (11.1)	8 (88.9)	0.46 [0.55–3.92]	0.44
Medication adherence (MMAS-4)^#^ score				
High adherence (0)	8 (22.9)	27 (77.1)		
Medium adherence (1-2)	9 (23.7)	29 (76.3)		
Low adherence (3-4)	21 (24.4)	65 (75.6)	1.64 [0.66–1.64]	0.85
Prevalence of comorbid condition	44 (25.4)	129 (74.6)	1.36 [0.59–3.18]	0.47
Individual comorbid conditions				
Diabetes mellitus	9 (23.1)	30 (76.9)	0.9 [0.40–2.10]	0.82
Kidney disease	7 (29.2)	17 (70.8)	1.32 [0.51–3.38]	0.57
Stroke	10 (50)	10 (50)	3.60 [1.40–9.21]	0.008
Heart failure	10 (18.5)	44 (81.5)	0.63 [0.29–1.36]	0.24

^#^MMAS-4, Morisky Medication Adherence Scale, the 4-item scale; IQR, interquartile range; CI, confidence interval.

**Table 4:** Factors associated with medication nonadherence by ordered logistic regression in 213 consecutive adults hospitalized to Bugando Medical Centre with hypertension-related diseases.

Variable	High adherence (MMAS; 0) *n* = 35 (%)	Medium adherence (MMAS; 1-2) *n* = 38 (%)	Low adherence (MMAS; 3-4) *n* = 86 (%)	Odds ratio [95% CI]	*p* value
Proportion (%)					
Median [IQR]					
Female gender	23 (28.1)	16 (19.5)	43 (52.4)	0.74 [0.41–1.34]	0.31
Age category					
≤45	12 (33.3)	9 (25.0)	15 (41.7)		
46–55	4 (12.9)	10 (32.3)	17 (54.8)		
56–65	11 (25.0)	7 (15.9)	26 (59.1)		
>65	8 (16.7)	12 (25.0)	28 (58.3)	1.24 [0.95–1.62]	0.12
Education level					
Not completed primary school	5 (11.9)	9 (21.4)	28 (66.7)		
Completed primary school	25 (27.5)	18 (19.8)	48 (52.8)		
Completed secondary school	5 (19.2)	11 (42.3)	10 (38.5)	0.63 [0.40–0.99]	0.04
Occupation					
Farmer	14 (25.9)	9 (16.7)	31 (57.4)		
Small scale business	5 (14.3)	10 (28.6)	20 (57.1)		
Professional, business, or student	16 (22.9)	19 (27.1)	35 (50.0)	1.20 [0.53–2.74]	0.68
No insurance	21 (21.2)	22 (22.2)	56 (56.6)	0.80 [0.44–1.48]	0.47
Systolic blood pressure					
≤140	8 (20.5)	14 (35.9)	17 (43.6)		
141–160	9 (20.5)	7 (15.9)	28 (63.6)	1.78 [0.78–4.08]	
161–180	7 (20.0)	8 (22.9)	20 (57.1)	1.45 [0.62–3.42]	
>180	11 (26.8)	9 (22.0)	21 (51.2)	1.09 [0.48–2.45]	0.51
Is hypertension curable (or is it a lifelong condition)?					
No (hypertension is a lifelong condition)	23 (28.8)	25 (31.3)	32 (40.0)		
Yes (hypertension is curable)	12 (15.2)	13 (16.5)	54 (68.4)	0.34 [0.18–0.63]	0.001
Is there anything that can be done to prevent hypertension?					
Yes	6 (12.8)	15 (31.9)	26 (55.3)		
No	29 (25.9)	23 (20.5)	60 (53.6)	1.30 [0.68–2.49]	0.43
Years of diagnosis					
1	12 (24.0)	10 (20)	28 (56.0)		
≥2	8 (19.1)	12 (28.6)	22 (52.4)		
≥5	8 (28.6)	4 (14.3)	16 (57.1)		
≥10	7 (18.0)	12 (30.8)	20 (51.3)	0.99 [0.77–1.28]	0.93
Place of diagnosis					
Hospital	35 (24.8)	34 (24.1)	72 (51.1)		
Other	0	4 (22.2)	14 (77.8)	3.80 [1.21–11.85]	0.02
Ever used herbs for hypertension	14 (20.6)	19 (27.9)	35 (51.5)	0.92 [0.50–1.67]	0.77
Ever used herbs for any reason	24 (20.7)	33 (28.5)	59 (50.9)	1.32 [0.72–2.67]	0.44
Ever attended traditional healer	24 (22.6)	28 (26.4)	54 (50.9)	1.36 [0.72–2.60]	0.35
Stopped allopathic medications for herbal medicine	7 (20.6)	6 (17.7)	21 (61.8)	0.72 [0.34–1.54]	0.40
Concurrent use of allopathic medications and herbal medicine	8 (21.1)	8 (21.1)	22 (57.9)	0.85 [0.42–1.72]	0.64
Believes it is okay to use herbal medicines with allopathic medicines	26 (2.6)	27 (23.5)	62 (53.9)	1.06 [0.54–2.05]	0.87
Prevalence of comorbid conditions	30 (22.2)	34 (25.2)	71 (52.6)	−0.33 [−1.20–0.55]	0.46
Individual comorbid conditions					
Diabetes mellitus	5 (15.2)	13 (39.4)	15 (45.5)	−0.14 [−0.4–0.56]	0.67
Kidney disease	6 (33.3)	1 (5.6)	11 (61.1)	0.01 [−0.1–1.02]	0.98
Stroke	2 (10.5)	5 (26.3)	12 (63.16)	0.52 [−0.43–0.47]	0.29
Heart failure	10 (21.3)	10 (21.3)	27 (57.5)	0.17 [−0.49–0.83]	0.61

MMAS-4 refers to Morisky Medication Adherence Scale, the 4-item scale; IQR, interquartile range; CI, confidence interval.

## Additional Points

See http://apps.who.int/classifications/icd10/browse/2010/en#/I10-I15.
